# Supplementary material for: Uptake and Patient Perspectives on Additional Testing for Novel Disease-Associated Genes: Lessons from a PAH Cohort
Source: Genes (Basel). 2021 Sep 28;12(10):1540. doi: 10.3390/genes12101540 (PMC8536181; doi:10.3390/genes12101540)
Supplement: Supplementary file 1 [file genes-12-01540-s001.zip › File S1. English letter PAH - Recontact procedure.pdf]

Dear sir, madam,

At the VU Medical Centre, you are or have been treated for pulmonary arterial hypertension at the Pulmonary Disease clinic.
RECONTACT DNA+, MUTATION-: Previously DNA-testing was performed to identify a genetic cause of pulmonary arterial hypertension. A genetic cause was not identified at that time. It is now possible to perform more extensive DNA testing
RECONTACT DNA-: To identify a potential genetic cause of pulmonary arterial hypertension, it is possible to perform DNA-testing at the Clinical Genetics Clinic of the VU Medical Centre.
In this letter, I would like to inform you about this possibility.

Current knowledge:
Recently, several additional genes associated with pulmonary arterial hypertension were identified.
RECONTACT DNA+, MUTATION-: Therefore, it is now possible that - while previous DNA-testing did not identify a genetic cause – a genetic cause can be identified. This chance is small. Nevertheless, you are eligible for additional DNA-testing to exclude any currently known genetic causes of pulmonary arterial hypertension.
RECONTACT DNA-: Therefore, it is possible to identify a genetic cause of pulmonary arterial hypertension by performing DNA-testing. In about 6% of patients with pulmonary arterial hypertension more than one person in the family is diagnosed with the disease. More and more often, it is possible to find a hereditary predisposition within these families using DNA diagnostics. Even if no other family members are known (yet) to have the disease, it is possible that a hereditary predisposition is identified. For this reason, each patient with idiopathic pulmonary arterial hypertension is eligible for DNA-testing.

If a gene mutation is found that is known to cause pulmonary arterial hypertension, close family members have a 50% chance of also carrying the hereditary predisposition for the disease. Family members subsequently can also be tested if desired. Carriers of the hereditary predisposition can regularly be checked by the lung specialist and, if necessary, treated in time. By detecting and treating the disease at an early stage, health benefits can be achieved. For more information on pulmonary arterial hypertension and genetics, an information letter is attached. You may also like to visit the website www.vumc/nl/afdelingen/ph-kenniscentrum.

How can you make an appointment for more information and/or DNA-testing?
On *** at *** AM/PM, you will be contact by phone to further explain this letter and answer any questions you may have. If you subsequently decide that you would like to undergo (more extensive) DNA-testing for the hereditary type of pulmonary arterial hypertension, ...

RECONTACT DNA +, MUTATION -: ... you can indicate this by filling out the enclosed application form. You can send the application form to VUmc, Clinical Genetics Department, Reception D, Antwoordnummer 7700, 1000 SN Amsterdam (no stamp needed), or send it by email it to klg.balie@vumc.nl . Because you previously had DNA-testing, the test can be done on DNA that has been stored. Therefore you do not have to visit the VU Medical Center. The result takes about two months. You will receive the results of the DNA-test by letter. If DNA testing reveals a hereditary predisposition for pulmonary hypertension, a consultation with the clinical geneticist will be scheduled to further inform you about the consequences for your family members and how you can inform them about this.
RECONTACT DNA -:. you can ask your general practitioner or lung specialist to refer you to the Clinical Genetics Clinic of the VU University Medical Center. To schedule an appointment, please fill out the enclosed registration form, signed and stamped by your general practitioner, or send it together with the referral letter from your general practitioner to VUmc, Clinical Genetics Clinic, Reception D, Antwoordnummer 7700, 1000 SN Amsterdam (no stamp needed), or mail it to klg.balie@vumc.nl.

I hope to have informed you sufficiently with this.

Sincerely,

LUNG SPECIALIST/CLINICAL GENETICIST

Attachments:
1. Information letter 'Pulmonary hypertension and genetics’.
2. Application form
