# Supplementary material for: Uptake and Patient Perspectives on Additional Testing for Novel Disease-Associated Genes: Lessons from a PAH Cohort
Source: Genes (Basel). 2021 Sep 28;12(10):1540. doi: 10.3390/genes12101540 (PMC8536181; doi:10.3390/genes12101540)
Supplement: Supplementary file 1 [file genes-12-01540-s001.zip › File S2. English letter PAH - Family letter relatives.pdf]

Dear sir, madam,

In your family, the hereditary lung disease pulmonary arterial hypertension has been diagnosed. The genetic cause was identified. It concerns the *** mutation in the *** gene. Brothers, sisters, parents and (minor) children of someone who carries a genetic predisposition for pulmonary arterial hypertension each have a 50% chance of carrying it. Family members who wish to do so can now have a DNA-test to find out whether they have an increased risk of contracting the disease. Children of deceased siblings of someone carrying the hereditary predisposition can also carry this predisposition, and are therefore also eligible for DNA-testing. If the parents of someone with a genetic predisposition passed away, family members of both parents (brothers, sisters or if they have died, their children) are also eligible.

It is thus important to try reach out to all family members with this letter so that they have the option to decide whether they wish to opt for DNA-testing on the hereditary predisposition found in the family. In the enclosed information letter and on our website (www. https://www.vumc.nl/zorg/expertisecentra-en-specialismen/pulmonale-hypertensie-kenniscentrum.htm.nl) you will find more information about the disease, the genetic cause and the possibilities and consequences of testing for it.

Characteristics, causes and inheritance of pulmonary arterial hypertension
For this I refer you to the attached information letter.

How do you make an appointment for more information and/or further testing?
If you have questions or would like to be tested on the hereditary predisposition for pulmonary arterial hypertension you can ask your general practitioner for a referral to a Clinical Genetics Clinic. To schedule an appointment, you can use the enclosed registration form, signed and stamped by your general practitioner, or mail or email it together with the referral letter from your physician (email address: ***). It is also possible to be referred to a Clinic Genetics Clinic near you. You will then be invited for a consultation with the clinical geneticist or genetic counsellor, and, if you wish, a DNA-test.

Psychosocial workers of the Clinical Genetics Clinic are always involved in the testing of underage children. You will speak with one of them by telephone prior to your appointment at the outpatient clinic. You can find more information about this on our website.

Information for the Clinical Genetics Outpatient Clinic
It is important that you bring the following information with you to the clinical genetics consultation.
FF number index patient: ***

DNA laboratory: ***
DNA family number: ***
Gene: ***
Mutation: ***

Costs
The costs for the informative consultation at the outpatient clinic and possible DNA diagnostics are generally covered by the basic health insurance (with the exception of some in-kind/budget insurances). You can check this in your insurance papers or ask your insurance company. How much you still have to pay depends on how high your deductible is and whether you have already used part of the deductible.

I hope to have informed you sufficiently with this.

Sincerely,

*** (CLINICAL GENETICIST)

Attachments:
1. Information letter about pulmonary arterial hypertension
2. Application form
